# Charge transport in germanium doped phosphorene nanoribbons

**DOI:** 10.1039/c8ra03041c

**Published:** 2018-05-29

**Authors:** Maryam Azizi, Badie Ghavami

**Affiliations:** School of Nanoscience, Institute for Research in Fundamental Sciences (IPM) P. O. Box: 19395-5531 Tehran Iran m.azizi@ipm.ir badie.ghavami@ipm.ir

## Abstract

New two dimensional structures containing phosphorus and germanium atoms are introduced for nanoelectronic applications. Under various bias voltages, electronic transport in the systems has been studied with the non-equilibrium Green’s function formalism. *I*–*V* characteristics have been extracted. The density of states (DOS) and transmission spectra, *T*(*E*,*V*_bias_), have been investigated and it was shown that charge transport occurs when the bias voltage reaches about 1 *V*. The negative differential resistance (NDR) appears in zigzag phosphorene nanoribbons (zPNRs) while it is completely suppressed after replacing edge phosphorus atoms with germanium ones. The calculated molecular projected self-consistent Hamiltonian (MPSH) shows that the spatial distribution of orbital levels has been affected by the electrodes. The studied structures have a band-gap of about 0.7 eV which absorbs light in the visible range and thus these structures could be interesting contenders for solar cells applications.

## Abstract 2

New two dimensional structures containing phosphorus and germanium atoms are introduced for nanoelectronic applications.

## Graphical abstract

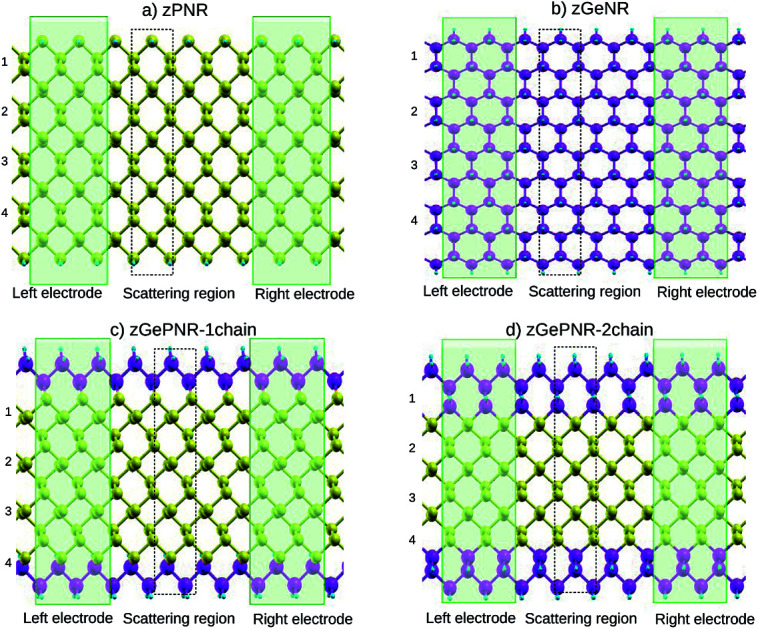

## 1. Introduction

Two dimensional (2D) graphene-like materials have recently attracted considerable attention due to their potential applications in nano- and optoelectronics.^1–4^ In particular, their unique size dependent properties allow for the exploration of a large number of novel phenomena at the nanoscale.^5–9^ Among 2D materials, the ones with sizable band-gaps are used in field effect transistor (FET) devices. In recent years, phosphorene^10–13^ (a monolayer of black phosphorus) has attracted great attention due to its reasonable mobility and band-gap which make it an attractive material for electronic applications. The effects of native defects,^14^ vacancies, and adatoms^15,16^ have been investigated for this 2D material. Moreover, the tuning of electronic properties, for example due to strain,^17–20^ paves the way to interesting applications of this material. In addition to phosphorene sheets, phosphorene nanoribbons (PNRs) have also been studied theoretically.^8,19,21^ Both armchair and zigzag PNRs show interesting optoelectrical and mechanical properties and have recently been under intense investigation by different scientific groups.

Another promising material with a tunable band-gap is germanene^22,23^ (a monolayer of germanium atoms). In a weak spin–orbit coupling regime, unique optical properties have been theoretically confirmed for germanene in the long wavelength limit.^24^ In the presence of an electric field, atoms in a buckled structure are no longer equivalent and a band-gap opening may happen in germanene. Therefore, germanene is of considerable interest for its application in field effect transistors at room temperature.^25^

Although both phosphorene and germanene nanoribbons show certain advantages in comparison to graphene and 2D dichalcogenides, such as their application in energy conversion/storage and high performance field effect transistors (FETs), there have been few transport studies on these materials in comparison to graphene and MoS_2_.^26–33^ Moreover, to our knowledge, the only recent work on the combination of these two elements^34^ considers the n layer of GeP_3_ and focuses on its low indirect band-gap and high carrier mobilities.

Since monolayers of germanium atoms show semimetallic properties, combining this element with phosphorus which is known as a wide band-gap material may result in the formation of a moderate band-gap material. Hence, in this paper, we introduce two nanoribbon structures composed of both germanium and phosphorus atoms. These new nanoribbons have a band-gap of about 0.7 eV which absorbs light in the visible range and could be suitable for solar cell applications. A non-equilibrium Green’s function method based on density functional theory (DFT) has been used to study the charge and quantum transport properties. The application of bias voltages, the density of states (DOS) and transmission spectra, as a function of energy, have been investigated.

The paper is organized as follows. In Section 2, we first introduce the system and explain the method which is used to derive the current–voltage characteristics, density of states (DOS) and transmission spectra. Then in Section 3, we present our numerical results. In particular, the role of bias voltages on the transmission spectrum has been shown. Finally, we conclude and summarize the main achievements in Section 4.

## 2. Model

### 2.1. Structure

**Fig. 1:**
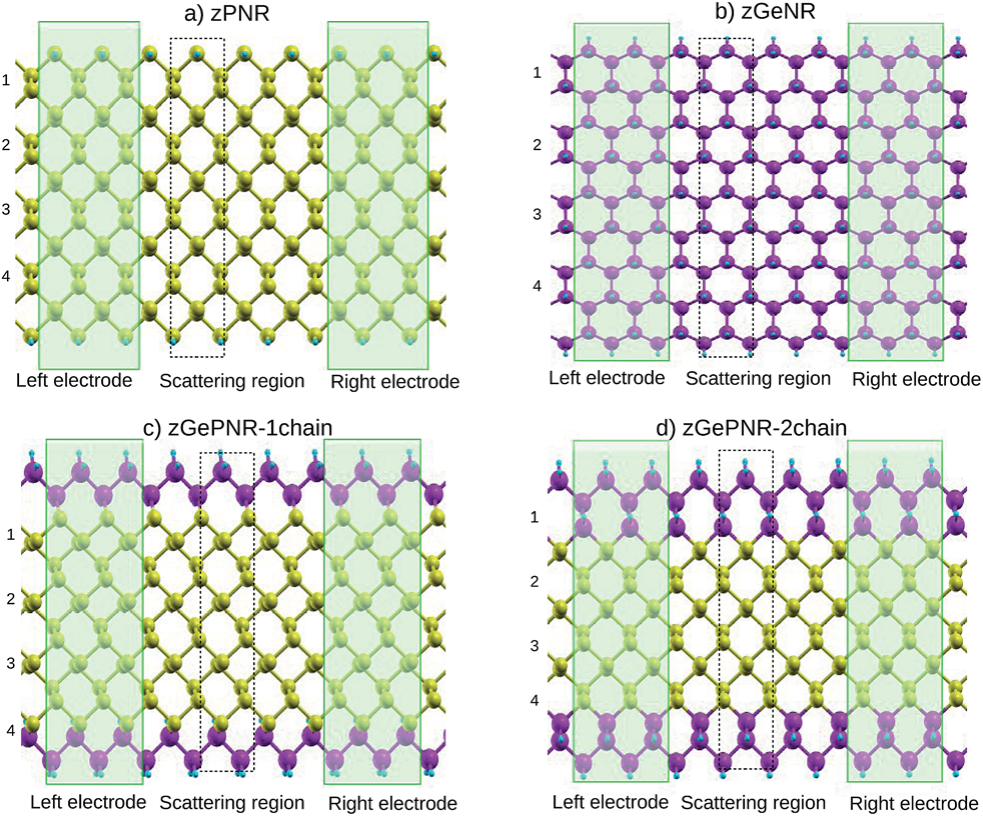
Schematic representation of (a) zPNR, (b) zGeNR, (c) zGePNR-1chain and (d) zGePNR-2chain. The green rectangles denote the semi-infinite left and right electrodes. One unit cell in the scattering region is indicated by a dashed rectangle. The numbers listed on the left side of the computational models indicate the width of the nanoribbons. Phosphorus, germanium and hydrogen are represented by yellow, purple and blue spheres, respectively.

In this work we study nanoribbon structures composed of germanium and phosphorus atoms, with a band-gap between pure germanene and pure phosphorene nanoribbons, as depicted in Fig. 1. In this model phosphorus atoms in both zigzag edges of the phosphorene nanoribbon, as shown in Fig. 1a, were replaced by germanium atoms to construct the structure depicted in Fig. 1c. To go further with the investigation of the effect of germanium dopants in zPNR, the second chains of phosphorus atoms were replaced by germanium ones, as shown in Fig. 1d. We assumed that each edge atom was passivated with enough H to remove dangling bonds.

This replacement decreases the band-gap of the pure phosphorene nanoribbon and makes it more suitable for the certain electronic applications discussed in Section 3.

### 2.2. Computational details

First principles calculations were performed using the *ab initio* Density Functional Theory (DFT) method, as implemented in the SIESTA open-source package^35^ which employs a linear combination of pseudoatomic orbitals as a basis set.

We assume infinite length and periodic boundary conditions in the *x*-direction. To avoid interactions between the adjacent layers, more than 20 Å vacuum space is considered to separate the nanoribbons in two other directions. Spin-unrestricted DFT calculations were performed using the Perdew–Burke–Ernzerhof (PBE) for generalized gradient approximation (GGA) exchange and correlation functional approach to obtain the band structures for the selected computational models.

In our calculations, a Brillouin zone is sampled with a 50 × 1 × 1 Monkhorst–Pack *k*-point grid and the cutoff energy is fixed to be 150 Ry.

Double-ζ polarized basis sets are used for the valence band electrons. Atomic and electronic degrees of freedom of the computational structures were allowed to relax simultaneously and self consistently until the interatomic forces were less than 0.002 eV/Å. The forces on ions were computed using a variant of the Hellmann–Feynman theorem^36,37^ that included Pulay type corrections to take into account the fact that the basis sets are not complete and move with the atoms.

In order to perform the calculations for charge transport and the electrical properties of the systems, non-equilibrium Green’s function (NEGF)^38^ equations were solved using Kohn–Sham wave functions obtained from DFT,^39^ as implemented in the TRANSIESTA open-source package^40^ at room temperature.

To investigate the transport properties, we specified three regions within the sample: two electrodes and the central (device) region (Fig. 1). The latter contained a portion of physical electrodes, the so-called right and left contacts, where all screening effects took place.

The transmission function of the system is calculated according to the following equation:^41^

1 *T*(*E*,*V*_b_) = Tr[*Γ*_L_(*E*,*V*_b_)*G*(*E*,*V*_b_)*Γ*_R_(*E*,*V*_b_)*G*^†^(*E*,*V*_b_)]

where, *E*, *V*_b_ and *G* are the energy, bias voltage and Green’s function, respectively. *Γ*_L(R)_ is the spectral density describing the coupling between the right (left) electrode and the scattering region.

In these structures current *vs.* bias voltage is extracted by the Landauer–Büttiker^42,43^ formula,2
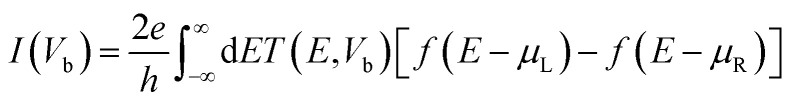
where, *μ*_R(L)_ is the chemical potential of the right (left) electrode, eV_b_ = *μ*_L_ − *μ*_R_ and *f*(*E* − *μ*_R(L)_) is the Fermi function.

We changed the bias voltage from 0.0 V to 2.0 V with a step of 0.05 V to achieve convergence of the density matrix.

## 3. Results and discussion

**Fig. 2:**
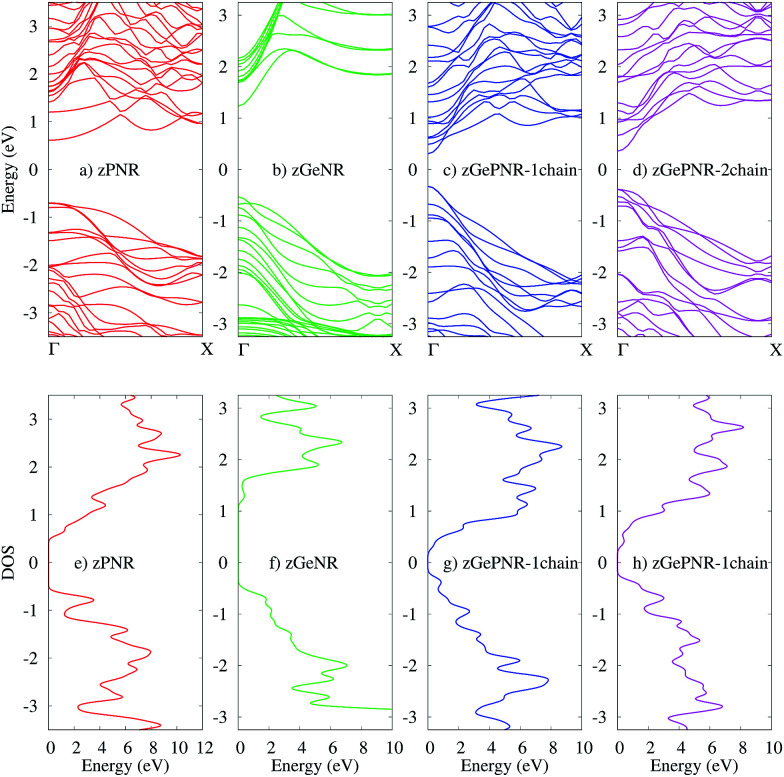
The band structures of (a) zPNR, (b) zGeNR, (c) zGePNR-1chain and (d) zGePNR-2chain and their corresponding DOS in (e), (f), (g) and (h), respectively.

The band structures of the studied configurations are illustrated in Fig. 2. In the case of phosphorene nanoribbons, each phosphorus atom is covalently bonded to three other P atoms and forms an sp^3^ hybridization to construct a puckered honeycomb structure with a DFT band-gap of about 1.3 eV. The chemical bonds in germanium are actually sp^3^-like^44^ for the partial hybridization of s and p_*Z*_ in Ge, but Ge has a larger covalent radius and its standard atomic weight is two times bigger than that of P. All of these specifications along with its electronegativity, which is almost of the same order as P, result in a DFT band-gap of 1.67 eV which is not very far from the phosphorene DFT band-gap.

The lower panels in Fig. 2 represent the density of states (DOS) for the bulk of all four nanoribbons studied in this paper. As shown in these graphs, zPNR (zigzag edge phosphorene nanoribbon) has a higher density of states in the region close to the Fermi energy (*E* = 0) than zGeNR (zigzag edge germanene nanoribbon). A higher DOS means more electrons (holes) in the conduction (valence) bands that could be followed by a smaller DFT band-gap in zPNR in comparison to zGeNR.

This is true for the two other configurations, zGePNR-1chain and zGePNR-2chain. Replacing the edge phosphorus atoms with germanium, as shown in Fig. 1c, leads to an increase in the DOS of zGePNR-1chain around the valence band maximum and conduction band minimum. As a result, the DFT band-gap decreases in comparison to those of pure zPNR and zGeNR (see Fig. 2). In order to explore the effect of increasing Ge dopants in phosphorene nanoribbons, we further replaced the next series of phosphorus atoms by Ge ones, as shown in Fig. 1d. zGePNR-2chain shows less DOS around the Fermi energy. This is the reason for zGePNR-2chain having a relatively larger DFT band-gap compared to zGePNR-1chain.

It should be noted that DFT-GGA typically underestimates the size of the band-gap compared to experimental values. However, the main idea of this work is to reveal the general trend of the zPNR band-gap in the presence of germanium atoms and the underlying physics of the new structures in the presence of an external electric field. Therefore, the underestimation of the band-gap does not affect our main conclusions.

**Fig. 3:**
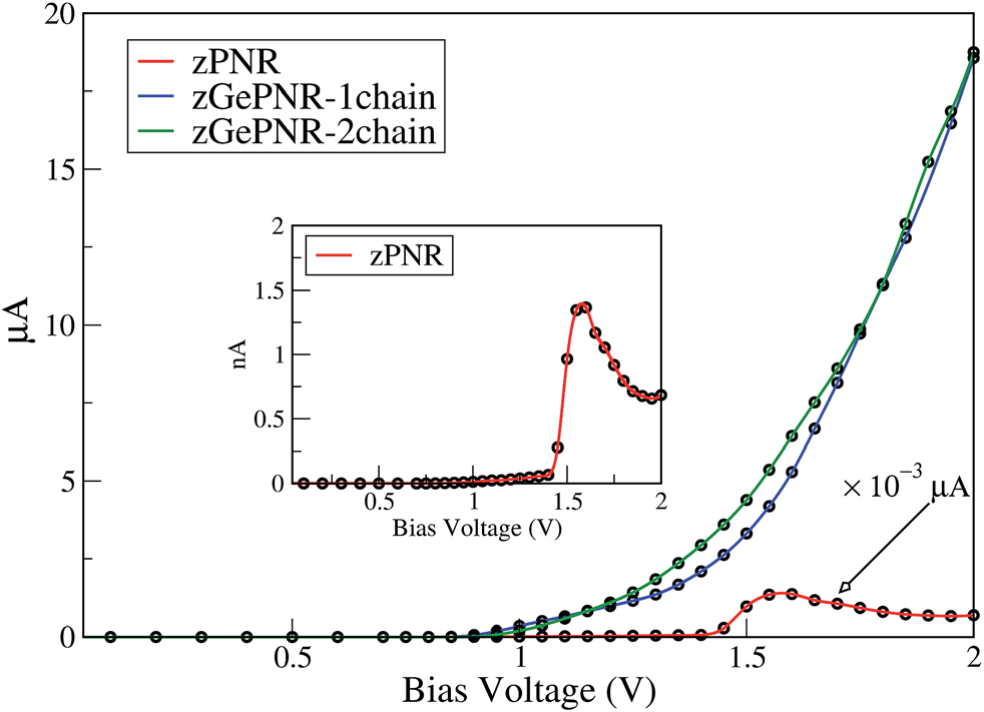
*I*–*V* characteristics of zPNR, zGePNR-1chain and zGeNPR-2chain.

To investigate the charge transport in our systems (containing electrodes and scattering regions), we determine the current–voltage bias characteristics of the configurations. As shown in Fig. 3, nonlinear *I*–*V*-trace characteristics for tunneling appear at an elevated bias.

It is noticeable that for bias voltages lower than 1 V the current nearly vanishes for the three systems while it increases with a relatively fast slope for higher bias voltages.

In fact, applying a bias voltage shifts the chemical potential of the left electrode with respect to the chemical potential of the right one. The current starts once the top of the valence band of the left electrode matches in energy with the bottom of the conduction band of the right electrode. This occurs in all of the systems, but for zPNR (inset in Fig. 3) it is about three orders of magnitude smaller than germanium doped zPNR and it rapidly increases at about 1.4 V.

The *I*–*V*_bias_ curve of germanium doped zPNR (1 and 2 chain) can be divided into three interesting regions. In the first one, up to 1.2 V both configurations (zGePNR-1chain and zGePNR-2chain) show the same behavior with almost the same current values. By increasing the bias voltage, they start to split with small differences up to 1.75 V. Then, again they behave in almost the same way.

**Fig. 4:**
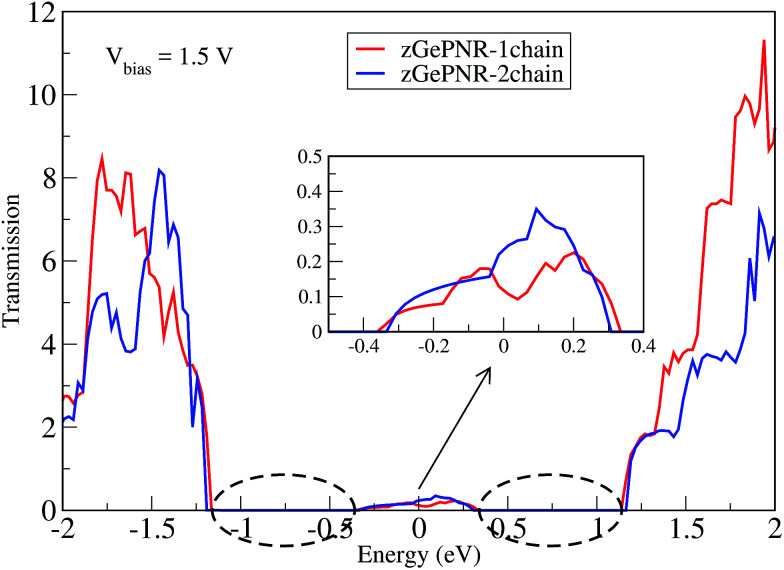
Transmission spectra of zGePNR-1chain and zGePNR-2chain at *V*_bias_ = 1.5 V. The dashed lines show the zero intervals of the transmission spectrum.

**Fig. 5:**
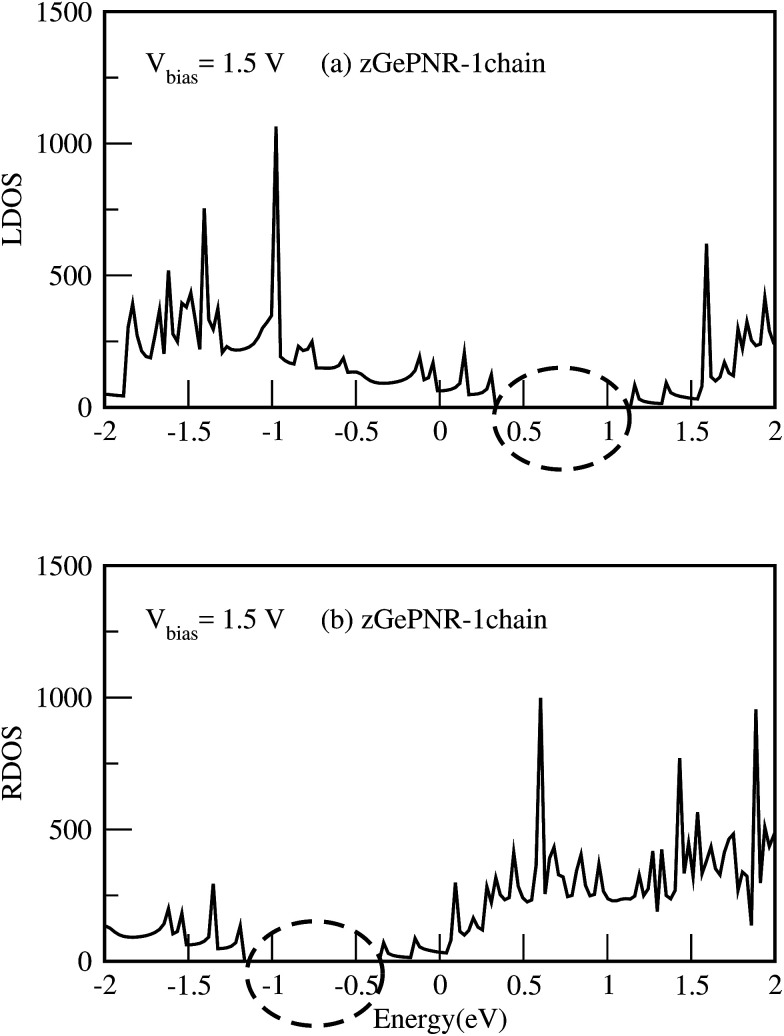
(a) LDOS and (b) RDOS of zGePNR-1chain at *V*_bias_ = 1.5 V. The dashed lines show the zero intervals of the LDOS and the RDOS.

This is confirmed in Fig. 4, where the transmission spectrum of both configurations is shown at *V*_bias_ = 1.5 V. To make it more clear, the left electrode DOS (LDOS) and the right electrode DOS (RDOS) have been illustrated in Fig. 5. As it is shown in this figure, some density of states (DOS) in the leads are suppressed by the screening which happens in the contact region. This is why the RDOS (LDOS) is zero in the energy interval of 1.16 eV to 0.36 eV (0.33 eV to 1.14 eV). From Fig. 4, it can be seen that the zero value of transmission happens exactly in the same intervals of energy, which results in no charge transfer from the leads to the scattering region (assigned by the dashed lines in Fig. 4 and 5).

The calculated DOS shows a peak around the Fermi level, which implies a strong correlation between the transmission and DOS. This occurs because transport at the Fermi level is dominated by resonant tunneling through interface states (see Fig. 5), not barrier tunneling.^45^ Under a positive bias voltage the chemical potential of the left (right) electrode shifts by eV/2(−eV/2). Therefore, the DOS of the left (right) electrode shifts toward a higher (lower) energy by eV/2, as shown in Fig. 5.

For the zPNR system, there is almost no carrier hopping between the electrodes and the scattering region when the bias range is limited below 1.4 V. The current increases with the applied bias voltage and reaches a maximum value of 1.36 nA at *V*_bias_ = 1.55 V. However, with a further increase in *V*_bias_, the current decreases dramatically, and consequently a negative differential resistance (NDR) phenomenon arises^8,46^ where the current decreases with an increment of voltage in some bias windows.

**Fig. 6:**
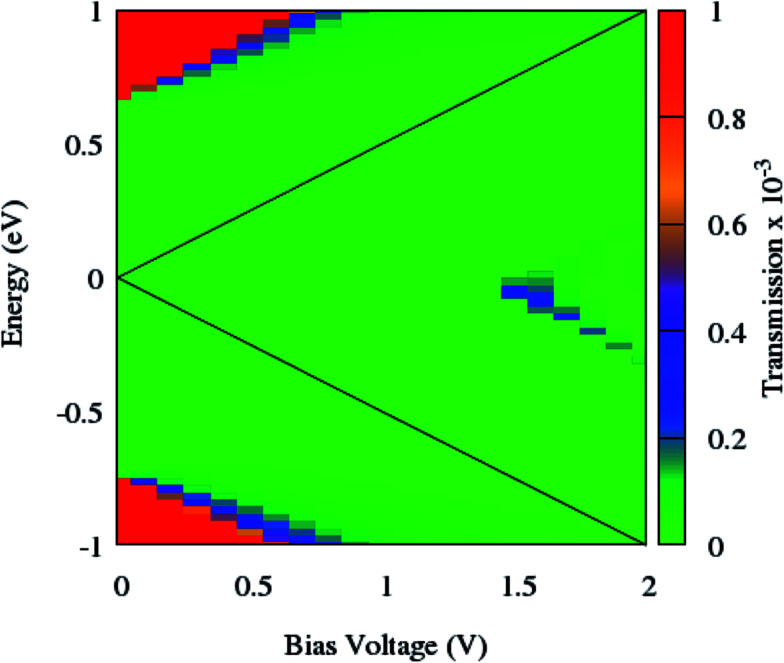
Contour map of the bias dependent transmission spectrum of zPNR. The black solid lines label the integral energy interval under an applied bias.

To clarify the NDR phenomenon in zPNR, the bias dependent transmission spectrum was checked and is depicted in Fig. 6 as a contour map and the black solid lines denote the integral energy interval under an applied bias. It can be clearly seen that there is almost no transmission up to *V*_bias_ = 1.4 V which means there is no charge transport in the system. Then the transmission starts increasing (moving from green to blue color) and the corresponding increase in current (Fig. 3) can be seen up to *V*_bias_ = 1.55 V which corresponds to the maximum value of current for zPNR. When the bias continuously increases, the transmission decreases to under 0.2 × 10^−3^ and the current never exceeds the peak value of 1.36 nA obtained at *V*_bias_ = 1.55 V (transition from blue to green color).

**Fig. 7:**
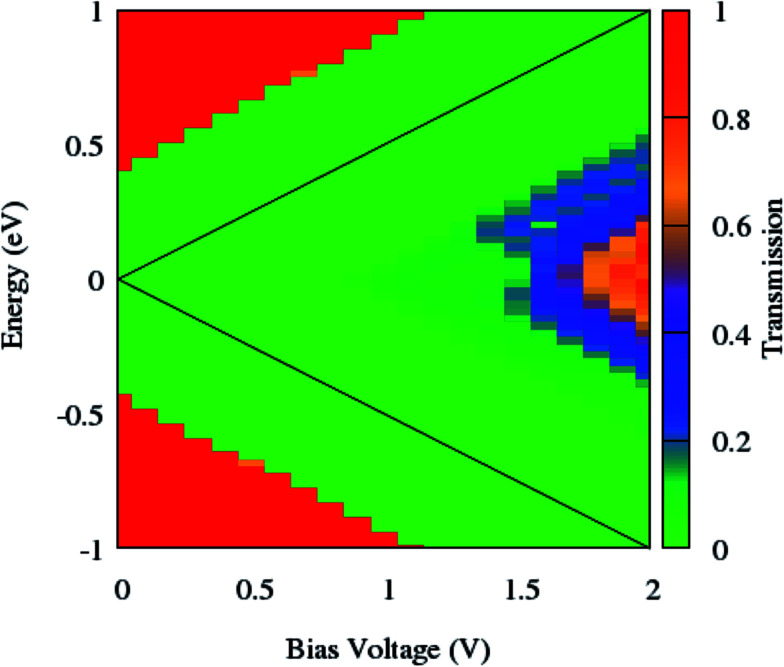
Contour map of the bias dependent transmission spectrum of zGePNR-1chain. The black solid lines label the integral energy interval under an applied bias.

As can be seen from Fig. 7, the NDR phenomenon is completely suppressed when the edge phosphorus atoms are replaced by germanium ones. The current continuously increases with the enhancement of the bias voltage and does not show any decreasing trend.

**Fig. 8:**
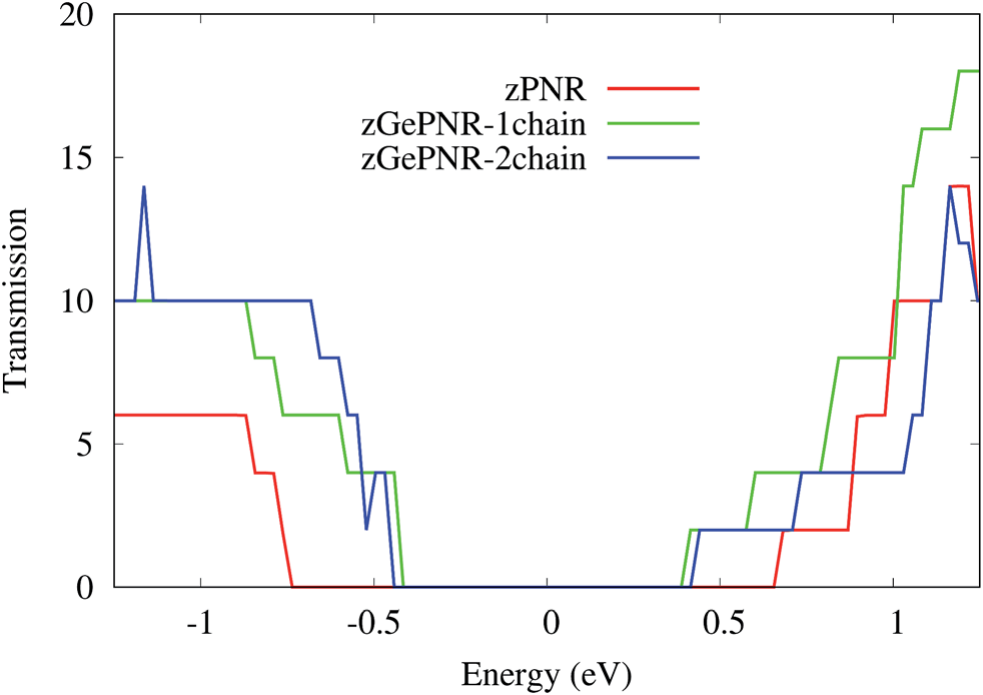
Transmission spectra for the three configurations in the absence of bias voltage.

According to Fig. 2, the band-gaps at zero bias voltage are of about 1.3 eV, 0.63 eV and 0.74 eV for zPNR, zGePNR-1chain and zGePNR-2chain, respectively, which shows that the presence of germanium decreases the band-gap and causes a corresponding increase in the transmission spectrum and the charge transport, as shown in Fig. 8. However, replacing the second chain of phosphorus atoms with Ge does not make a significant difference in the characteristics of the nanoribbons.

As is shown in Fig. 8, in the absence of bias voltage, both electrodes have relatively similar DOS, and hence the transmission function can be large enough wherever the electrodes have electronic states. However, since the bias window is zero, there is no current, as is confirmed by Fig. 3.

**Fig. 9:**
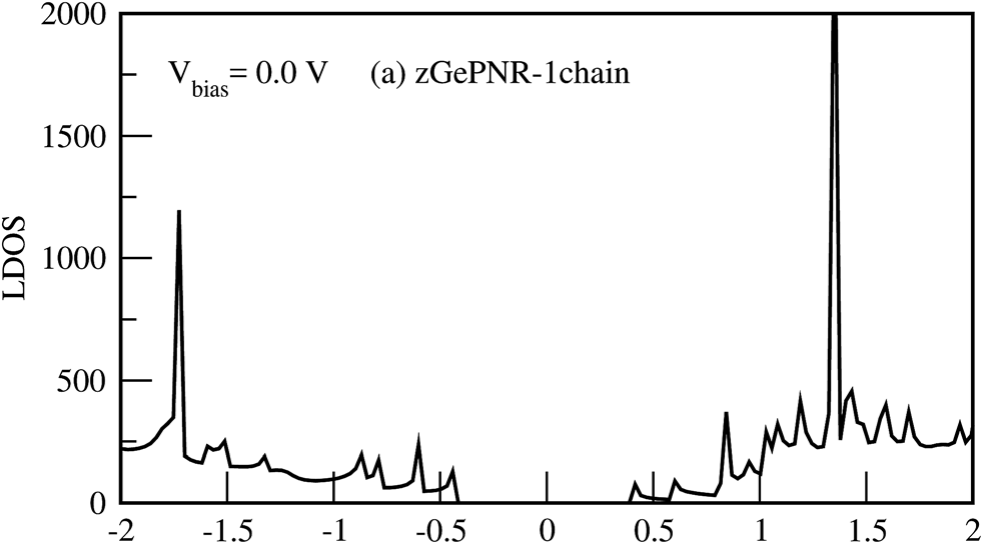
RDOS (LDOS) at *V*_bias_ = 0 V.

Fig. 9 shows the LDOS for zGePNR-1chain at *V*_bias_ = 0. Since there is no bias voltage, RDOS (not shown here) is exactly the same as LDOS. Accordingly, no overlap exists between the electrodes in the absence of bias voltage which means that there is no charge transfer.

**Fig. 10:**
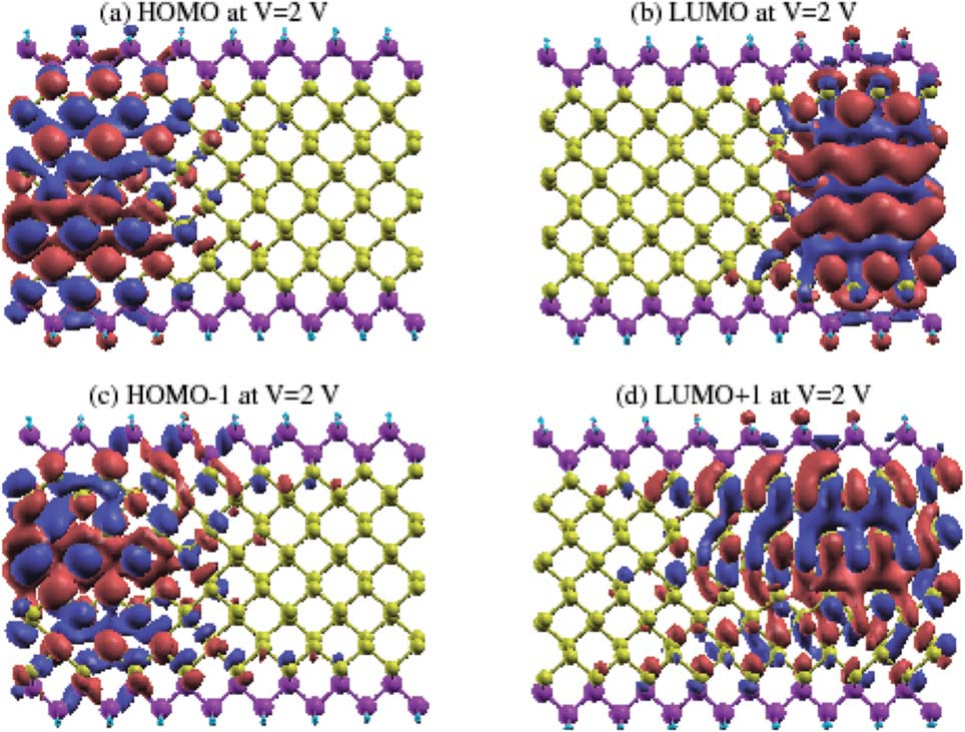
The HOMO, LUMO, HOMO−1 and LUMO+1 MPSH orbitals for zGeRNR-1chain at *V*_bias_ = 2 V.

In order to gain a better understanding of the effect of *V*_bias_, we show the eigenstates of the molecules placed in two different probe environments. The eigenvalues were calculated using the molecular projected self-consistent Hamiltonian (MPSH), as shown in Fig. 10, as implemented by the INELASTICA package^47–50^ to project scattering matrices onto localised eigenstates in order to differentiate between the conducting and non-conducting eigenstates.

The clearly non-zero transmission spectra emerges around the Fermi level and isosurfaces appear inside the device. Fig. 10 shows the highest occupied molecular orbital (HOMO) and lowest unoccupied molecular orbital (LUMO) for the first (Fig. 10a and b) and second (Fig. 10c and d) molecular orbital levels for zGePNR-1chain at *V*_bias_ = 2 V. The state overlap can be clearly seen between the HOMO−1 and LUMO+1 orbitals and strongly couples the left and right electrodes to the scattering region. Hence, the non-zero current appears from the left electrode and moves to the right one (Fig. 3 shows 4.4 μA). This is what is expected in a sizable band-gap semiconductor. The applied bias voltage changes the eigenchannels in the electrode–device interface and band-gap narrowing occurs due to the induced electrostatic potential across the nanoribbons.

**Fig. 11:**
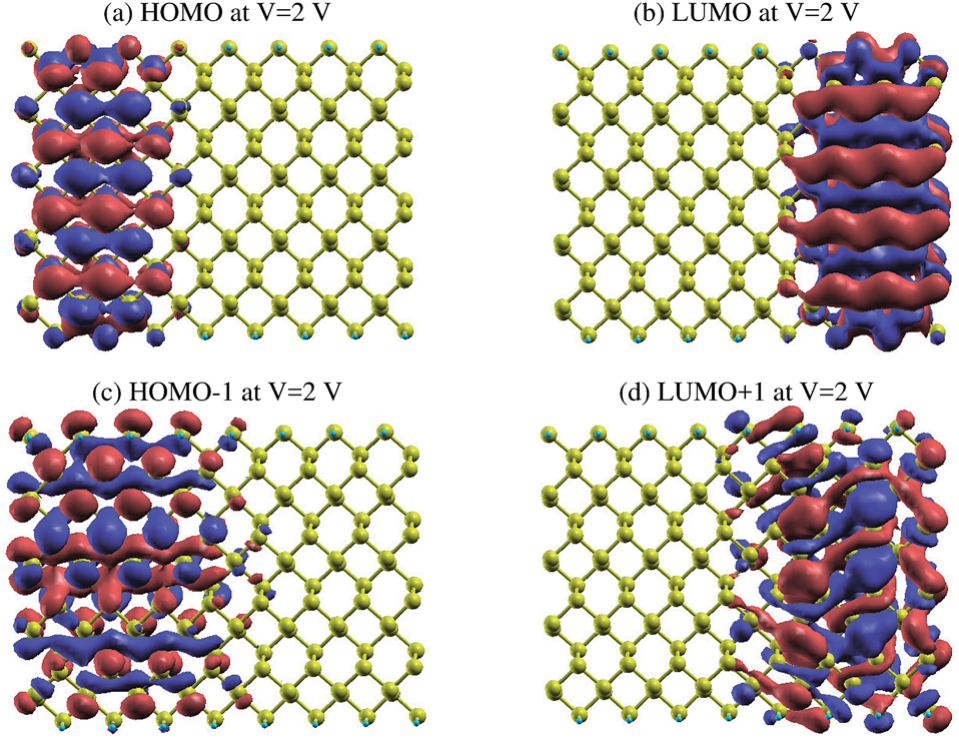
The HOMO, LUMO, HOMO−1 and LUMO+1 MPSH orbitals for zPNR at *V*_bias_ = 2 V.

The same effect occurs for zPNR, as shown in Fig. 11. The only difference is that for zPNR, the maximum amplitude of current is about three orders of magnitude smaller (in the order of nA). It is clear that for zPNR even at *V*_bias_ = 2 V, the highest applied bias, the left electrode is almost decoupled from the rest of the structure which corresponds to weak conductivity.

This is again a confirmation of the results shown in Fig. 5 that emphasizes that if Ge atoms are embedded in the structure, the spectra penetrate deeper into the conduction region compared to those for zPNR. This means Ge doped phosphorene nanoribbons are materials with smaller bad-gaps and could be better candidates for charge transport systems.

## 4. Conclusions

In summary, the *I*–*V* curves of two new systems consisting of phosphorus, germanium and hydrogen atoms in zigzag nanoribbons were examined using first-principles calculations. We have traced the current pathway by studying the transmission spectrum for different bias voltages.

First, the electronic structures of pure zPNR and zGeNR were analyzed. Then, we showed how replacing edge phosphorus atoms with germanium results in a decrease of the band-gap of zPNR.

DOS and *T*(*E*,*V*_bias_) were investigated and it was shown that the charge transport occurs when the bias voltage reaches to about 1 V. The transport channels were studied *via* the calculations of the current density and local electron transmission pathway.

The calculated MPSH shows that the spatial distribution of orbital levels was affected by the electrodes. The visualized transmission pathways show how charge carriers propagate through the scattering region in all of the systems.

The characteristics of the new structures were compared to those of zPNR. The results confirm that the band-gap of phosphorene nanoribbons in the presence of germanium decreases which results in an increase in charge transport.

The studied structure has a band-gap of about 0.7 eV which absorbs light in the visible range and thus is an interesting contender for solar cells and is suitable for practical applications in nanoelectronic and optoelectronic devices.

Finally, our calculations show that negative differential resistivity behavior in the zPNR device vanishes when the edge phosphorus atoms are substituted with Ge ones.

## Conflicts of interest

There are no conflicts to declare.

## Supplementary Material
